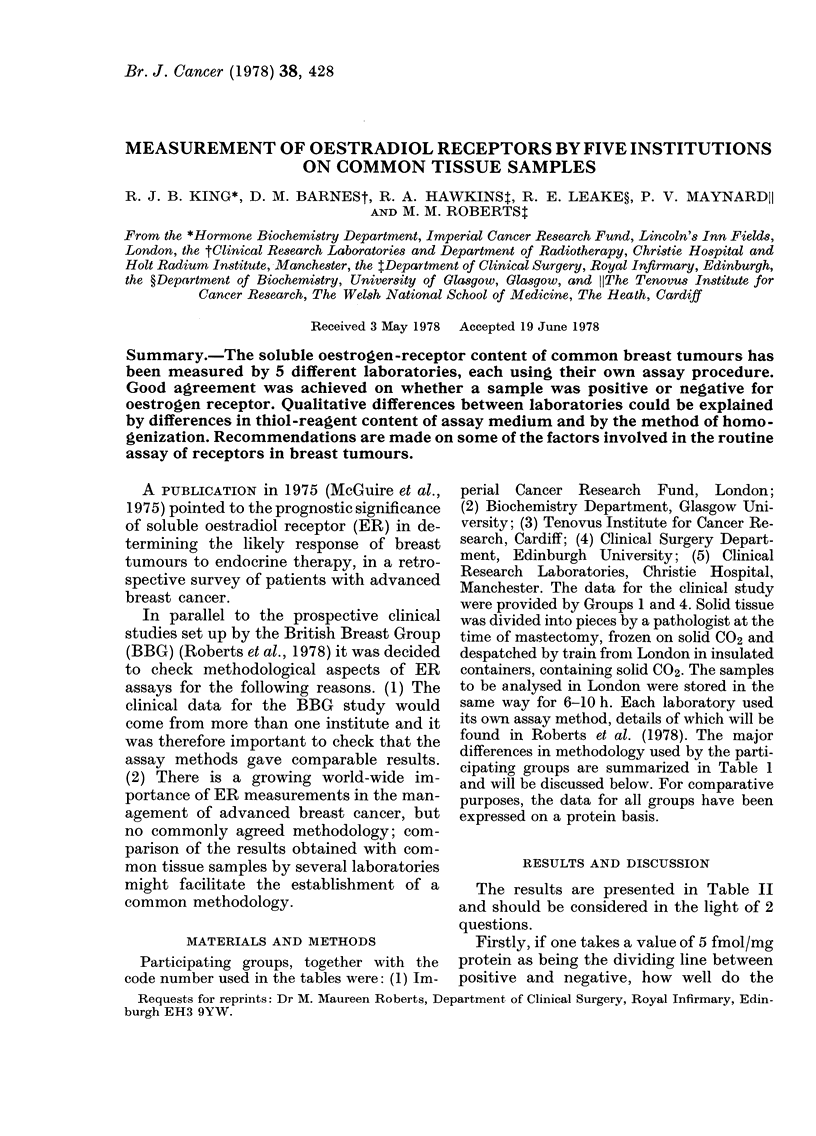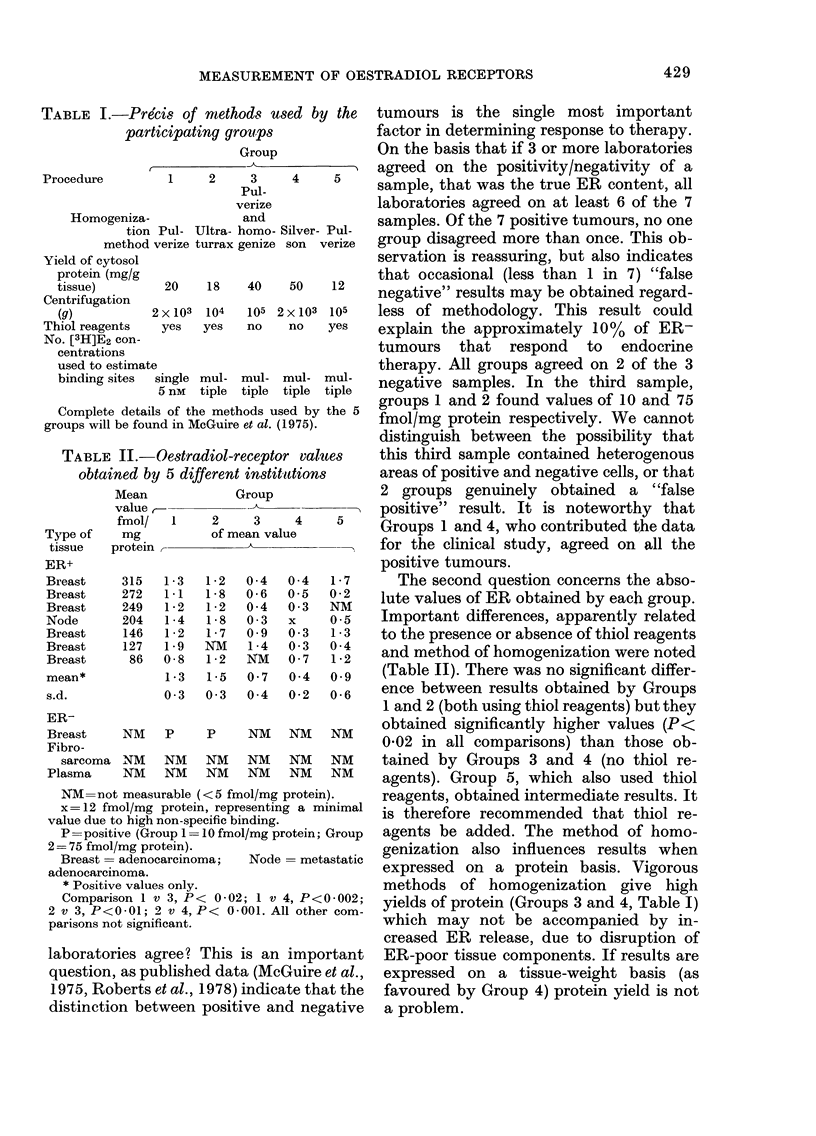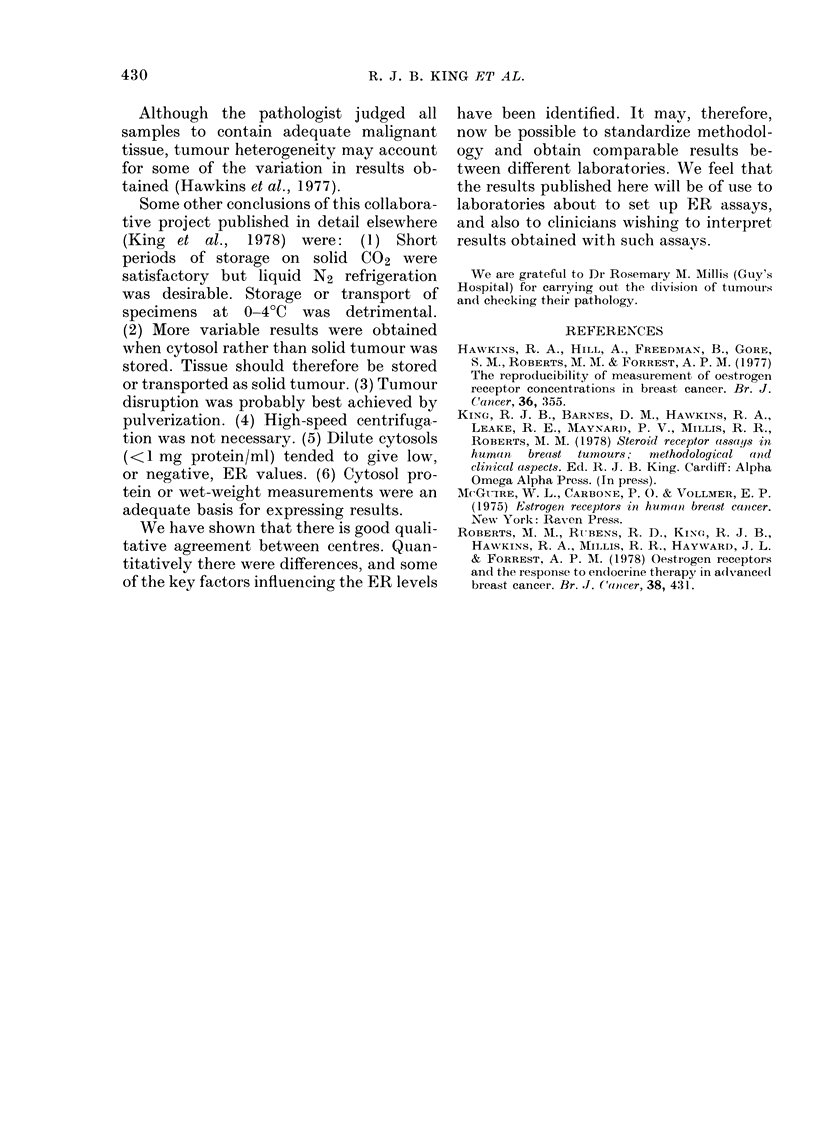# Measurement of oestradiol receptors by five institutions on common tissue samples.

**DOI:** 10.1038/bjc.1978.224

**Published:** 1978-09

**Authors:** R. J. King, D. M. Barnes, R. A. Hawkins, R. E. Leake, P. V. Maynard, M. M. Roberts

## Abstract

The soluble oestrogen-receptor content of common breast tumours has been measured by 5 different laboratories, each using their own assay procedure. Good agreement was achieved on whether a sample was positive or negative for oestrogen receptor. Qualitative differences between laboratories could be explained by differences in thiol-reagent content of assay medium and by the method of homogenization. Recommendations are made on some of the factors involved in the routine assay of receptors in breast tumours.


*Br. J. Cancer* (1978) **38**, 428

### MEASUREMENT OF OESTRADIOL RECEPTORS BY FIVE INSTITUTIONS ON COMMON TISSUE SAMPLES

R. J. B. KING*, D. M. BARNES†, R. A. HAWKINS‡, R. E. LEAKE§, P. V. MAYNARD|| AND M. M. ROBERTS‡

*From the *Hormone Biochemistry Department, Imperial Cancer Research Fund, Lincoln's Inn Fields, London, the †Clinical Research Laboratories and Department of Radiotherapy, Christie Hospital and Holt Radium Institute, Manchester, the ‡Department of Clinical Surgery, Royal Infirmary, Edinburgh, the §Department of Biochemistry, University of Glasgow, Glasgow, and ||The Tenovus Institute for Cancer Research, The Welsh National School of Medicine, The Heath, Cardiff*

Received 3 May 1978 Accepted 19 June 1978

**Summary.—The soluble oestrogen-receptor content of common breast tumours has been measured by 5 different laboratories, each using their own assay procedure. Good agreement was achieved on whether a sample was positive or negative for oestrogen receptor. Qualitative differences between laboratories could be explained by differences in thiol-reagent content of assay medium and by the method of homogenization. Recommendations are made on some of the factors involved in the routine assay of receptors in breast tumours.**

A PUBLICATION in 1975 (McGuire *et al.*, 1975) pointed to the prognostic significance of soluble oestradiol receptor (ER) in determining the likely response of breast tumours to endocrine therapy, in a retrospective survey of patients with advanced breast cancer.

In parallel to the prospective clinical studies set up by the British Breast Group (BBG) (Roberts *et al.*, 1978) it was decided to check methodological aspects of ER assays for the following reasons. (1) The clinical data for the BBG study would come from more than one institute and it was therefore important to check that the assay methods gave comparable results. (2) There is a growing world-wide importance of ER measurements in the management of advanced breast cancer, but no commonly agreed methodology; comparison of the results obtained with common tissue samples by several laboratories might facilitate the establishment of a common methodology.

### MATERIALS AND METHODS

Participating groups, together with the code number used in the tables were: (1) Imperial Cancer Research Fund, London; (2) Biochemistry Department, Glasgow University; (3) Tenovus Institute for Cancer Research, Cardiff; (4) Clinical Surgery Department, Edinburgh University; (5) Clinical Research Laboratories, Christie Hospital, Manchester. The data for the clinical study were provided by Groups 1 and 4. Solid tissue was divided into pieces by a pathologist at the time of mastectomy, frozen on solid $CO_2$ and despatched by train from London in insulated containers, containing solid $CO_2$. The samples to be analysed in London were stored in the same way for 6–10 h. Each laboratory used its own assay method, details of which will be found in Roberts *et al.* (1978). The major differences in methodology used by the participating groups are summarized in Table 1 and will be discussed below. For comparative purposes, the data for all groups have been expressed on a protein basis.

### RESULTS AND DISCUSSION

The results are presented in Table II and should be considered in the light of 2 questions.

Firstly, if one takes a value of 5 fmol/mg protein as being the dividing line between positive and negative, how well do the

Requests for reprints: Dr M. Maureen Roberts, Department of Clinical Surgery, Royal Infirmary, Edinburgh EH3 9YW.

TABLE I.—*Précis of methods used by the participating groups*

| Procedure | Group 1 | 2 | 3 | 4 | 5 |
|---|---|---|---|---|---|
| Homogenization method | Pulverize | Ultra-turrax | Pulverize and homogenize | Silverson | Pulverize |
| Yield of cytosol protein (mg/g tissue) | 20 | 18 | 40 | 50 | 12 |
| Centrifugation (*g*) | $2\times10^3$ | $10^4$ | $10^5$ | $2\times10^3$ | $10^5$ |
| Thiol reagents | yes | yes | no | no | yes |
| No. $[^3H]E_2$ concentrations used to estimate binding sites | single 5 nM | multiple | multiple | multiple | multiple |

Complete details of the methods used by the 5 groups will be found in McGuire *et al.* (1975).

TABLE II.—*Oestradiol-receptor values obtained by 5 different institutions*

| Type of tissue | Mean value fmol/mg protein | Group 1 | 2 | 3 | 4 | 5 |
|---|---|---|---|---|---|---|
| | | of mean value | | | | |
| $ER^+$ | | | | | | |
| Breast | 315 | 1·3 | 1·2 | 0·4 | 0·4 | 1·7 |
| Breast | 272 | 1·1 | 1·8 | 0·6 | 0·5 | 0·2 |
| Breast | 249 | 1·2 | 1·2 | 0·4 | 0·3 | NM |
| Node | 204 | 1·4 | 1·8 | 0·3 | x | 0·5 |
| Breast | 146 | 1·2 | 1·7 | 0·9 | 0·3 | 1·3 |
| Breast | 127 | 1·9 | NM | 1·4 | 0·3 | 0·4 |
| Breast | 86 | 0·8 | 1·2 | NM | 0·7 | 1·2 |
| mean* | | 1·3 | 1·5 | 0·7 | 0·4 | 0·9 |
| s.d. | | 0·3 | 0·3 | 0·4 | 0·2 | 0·6 |
| $ER^-$ | | | | | | |
| Breast | NM | P | P | NM | NM | NM |
| Fibrosarcoma | NM | NM | NM | NM | NM | NM |
| Plasma | NM | NM | NM | NM | NM | NM |

NM = not measurable (<5 fmol/mg protein).

x = 12 fmol/mg protein, representing a minimal value due to high non-specific binding.

P = positive (Group 1 = 10 fmol/mg protein; Group 2 = 75 fmol/mg protein).

Breast = adenocarcinoma; Node = metastatic adenocarcinoma.

* Positive values only.

Comparison 1 *v* 3, $P < 0·02$; 1 *v* 4, $P < 0·002$; 2 *v* 3, $P < 0·01$; 2 *v* 4, $P < 0·001$. All other comparisons not significant.

laboratories agree? This is an important question, as published data (McGuire *et al.*, 1975, Roberts *et al.*, 1978) indicate that the distinction between positive and negative tumours is the single most important factor in determining response to therapy. On the basis that if 3 or more laboratories agreed on the positivity/negativity of a sample, that was the true ER content, all laboratories agreed on at least 6 of the 7 samples. Of the 7 positive tumours, no one group disagreed more than once. This observation is reassuring, but also indicates that occasional (less than 1 in 7) "false negative" results may be obtained regardless of methodology. This result could explain the approximately 10% of $ER^-$ tumours that respond to endocrine therapy. All groups agreed on 2 of the 3 negative samples. In the third sample, groups 1 and 2 found values of 10 and 75 fmol/mg protein respectively. We cannot distinguish between the possibility that this third sample contained heterogenous areas of positive and negative cells, or that 2 groups genuinely obtained a "false positive" result. It is noteworthy that Groups 1 and 4, who contributed the data for the clinical study, agreed on all the positive tumours.

The second question concerns the absolute values of ER obtained by each group. Important differences, apparently related to the presence or absence of thiol reagents and method of homogenization were noted (Table II). There was no significant difference between results obtained by Groups 1 and 2 (both using thiol reagents) but they obtained significantly higher values ($P <$ 0·02 in all comparisons) than those obtained by Groups 3 and 4 (no thiol reagents). Group 5, which also used thiol reagents, obtained intermediate results. It is therefore recommended that thiol reagents be added. The method of homogenization also influences results when expressed on a protein basis. Vigorous methods of homogenization give high yields of protein (Groups 3 and 4, Table I) which may not be accompanied by increased ER release, due to disruption of ER-poor tissue components. If results are expressed on a tissue-weight basis (as favoured by Group 4) protein yield is not a problem.

Although the pathologist judged all samples to contain adequate malignant tissue, tumour heterogeneity may account for some of the variation in results obtained (Hawkins *et al.*, 1977).

Some other conclusions of this collaborative project published in detail elsewhere (King *et al.*, 1978) were: (1) Short periods of storage on solid $CO_2$ were satisfactory but liquid $N_2$ refrigeration was desirable. Storage or transport of specimens at 0–4°C was detrimental. (2) More variable results were obtained when cytosol rather than solid tumour was stored. Tissue should therefore be stored or transported as solid tumour. (3) Tumour disruption was probably best achieved by pulverization. (4) High-speed centrifugation was not necessary. (5) Dilute cytosols (<1 mg protein/ml) tended to give low, or negative, ER values. (6) Cytosol protein or wet-weight measurements were an adequate basis for expressing results.

We have shown that there is good qualitative agreement between centres. Quantitatively there were differences, and some of the key factors influencing the ER levels have been identified. It may, therefore, now be possible to standardize methodology and obtain comparable results between different laboratories. We feel that the results published here will be of use to laboratories about to set up ER assays, and also to clinicians wishing to interpret results obtained with such assays.

We are grateful to Dr Rosemary M. Millis (Guy's Hospital) for carrying out the division of tumours and checking their pathology.

### REFERENCES

HAWKINS, R. A., HILL, A., FREEDMAN, B., GORE, S. M., ROBERTS, M. M. & FORREST, A. P. M. (1977) The reproducibility of measurement of oestrogen receptor concentrations in breast cancer. *Br. J. Cancer*, **36**, 355.

KING, R. J. B., BARNES, D. M., HAWKINS, R. A., LEAKE, R. E., MAYNARD, P. V., MILLIS, R. R., ROBERTS, M. M. (1978) *Steroid receptor assays in human breast tumours; methodological and clinical aspects*. Ed. R. J. B. King. Cardiff: Alpha Omega Alpha Press. (In press).

MCGUIRE, W. L., CARBONE, P. O. & VOLLMER, E. P. (1975) *Estrogen receptors in human breast cancer*. New York: Raven Press.

ROBERTS, M. M., RUBENS, R. D., KING, R. J. B., HAWKINS, R. A., MILLIS, R. R., HAYWARD, J. L. & FORREST, A. P. M. (1978) Oestrogen receptors and the response to endocrine therapy in advanced breast cancer. *Br. J. Cancer*, **38**, 431.